# Mycotoxins in Food and Feeds: Human Health and Animal Nutrition

**DOI:** 10.3390/toxins18070297

**Published:** 2026-07-10

**Authors:** María Agustina Pavicich, Andrea Patriarca

**Affiliations:** 1 Centre of Excellence in Mycotoxicology and Public Health, Department of Bioanalysis, Faculty of Pharmaceutical Sciences, Ghent University, 9000 Ghent, Belgium; 2 Magan Centre of Applied Mycology, Cranfield University, College Road, Bedford MK43 0AL, UK

The landscape of mycotoxin research is evolving rapidly. This Special Issue was conceived to capture these developments, bringing together studies that span analytical chemistry, toxicology, exposure science, computational modelling, food processing, intervention strategies and new methodologies. Collectively, the contributions illustrate how modern mycotoxin research is moving beyond monitoring contamination towards a more integrated understanding of exposure, risk prediction, and mitigation.

This evolution is occurring at a particularly important time. Global food systems are being reshaped by climate change, changing agricultural practices, and increasingly interconnected supply chains, all of which influence the distribution of toxigenic fungi and the occurrence of both established and emerging mycotoxins. Alongside well-known contaminants such as aflatoxins and ochratoxin A, increasing attention is being directed towards less-studied compounds, including *Alternaria* toxins, whose occurrence, toxicology, and health implications remain incompletely understood. Addressing these emerging challenges requires multidisciplinary research that integrates analytical innovation with toxicology, exposure science, predictive modelling, and practical mitigation strategies.

The nine papers gathered in this Special Issue reflect this changing landscape. Rather than focusing on a single aspect of mycotoxin research, they collectively illustrate how the field is moving towards more predictive, data-driven and prevention-oriented approaches that ultimately support safer food and feed systems.

Surveillance studies remain fundamental for food safety. Nandinidevi et al. [1] investigated ochratoxin A contamination and *Aspergillus* spp. in brown and polished rice from Indian markets, emphasizing the need for continued monitoring and improved storage practices. Understanding toxicity is equally essential. Mubarik et al. [2] systematically reviewed the effects of ochratoxin A on innate and adaptive immunity, summarizing growing evidence that OTA disrupts immune homeostasis through multiple molecular pathways.

Visintin et al. [3] derived human toxicokinetic parameters for citrinin using an intervention study combined with hierarchical Bayesian modelling, providing data directly relevant for human risk assessment, demonstrating that improving exposure assessment remains a major priority. Complementing this work, Maris et al. [4] demonstrated how human biomonitoring can evaluate the effectiveness of nixtamalization in reducing mycotoxin exposure from maize under real-world conditions. Computational toxicology is also becoming increasingly important. Lootens et al. [5] developed physiologically based pharmacokinetic populations for Sub-Saharan Africa, revealing critical data gaps that currently limit accurate regional exposure assessment for aflatoxin B1.

Among emerging mycotoxins, *Alternaria* toxins have received increasing attention over the last decade owing to their widespread occurrence in fruits, vegetables and cereal products, coupled with important gaps in our understanding of exposure and toxicity. Two contributions to this Special Issue address complementary aspects of this challenge: untargeted LC-HRMS metabolomics for the detection of *Alternaria*-infected apples and the development of a validated LC-MS/MS method for determining *Alternaria* toxins in hepatic tissue [6,7]. Together, these studies illustrate how advances in analytical science are expanding our capacity to investigate emerging mycotoxins from food contamination through biological exposure.

Advances in non-destructive analytical technologies continue to transform mycotoxin detection. Kabir et al. [8] reviewed recent developments in hyperspectral imaging integrated with machine learning for detecting mycotoxins in cereals and nuts, highlighting the opportunities and remaining challenges for industrial implementation. Building on these concepts, the same group subsequently developed an attention-guided three-dimensional deep learning architecture (AGIR-3DNet) capable of rapidly detecting aflatoxin B1 contamination in almonds while substantially improving computational efficiency, bringing hyperspectral imaging one step closer to real-time industrial applications [9].

Looking ahead, addressing the global challenge of mycotoxins will require continued collaboration among researchers, industry, regulatory authorities, and policymakers. Integrating advances in analytical science, toxicology, predictive modelling, crop management, and food systems will be essential to developing sustainable solutions that safeguard both human and animal health. A One Health perspective, recognizing the interconnectedness of agricultural production, environmental change, food safety, and health, provides an appropriate framework for tackling these complex challenges.

Collectively, the studies published in this Special Issue demonstrate that mycotoxin research is entering a new phase. The convergence of advanced analytical technologies, artificial intelligence, computational modelling, human biomonitoring and intervention studies is transforming our ability not only to detect contamination, but also to understand exposure, predict risk and develop effective mitigation strategies. As new challenges emerge, including climate-driven changes in fungal ecology and the increasing recognition of emerging mycotoxins, continued interdisciplinary collaboration will be essential to ensure that scientific advances translate into improved food and feed safety policies.

As Guest Editors, we sincerely thank all the authors who contributed their high-quality research to this Special Issue. Their work demonstrates the breadth and vitality of contemporary mycotoxin research and advances our collective understanding of this important field. We are equally grateful to the reviewers for their time, expertise, and constructive comments, which ensured the scientific quality of the published articles. Finally, we thank the Editorial Office of Toxins for their professional support throughout the preparation of this Special Issue.

It is our hope that this Special Issue will not only serve as a snapshot of current progress but also encourage future research that is increasingly predictive, collaborative and internationally connected. By integrating analytical innovation with toxicological understanding and risk assessment, the mycotoxin research community will be better equipped to protect both human and animal health in an increasingly dynamic global food system.
